# Interplay between evolutionary history, morphological constraints and functional adaptations in the primate cochlea

**DOI:** 10.1098/rsos.250802

**Published:** 2025-11-05

**Authors:** Joaquin del Rio, Manuela Nowotny, Romain David, Alexander Stoessel

**Affiliations:** ^1^ Department of Archaeogenetics, Max Planck Institute for Evolutionary Anthropology, Leipzig, Germany; ^2^ Institute for Zoology and Evolutionary Research, Friedrich Schiller University Jena, Jena, Germany; ^3^ Centre for Human Evolution Research, Natural History Museum, London, UK

**Keywords:** primates, cochlea, evolution, geometric morphometrics, ancestral state reconstruction, shape

## Abstract

How the intricate mammalian cochlea evolved, and its functional implications, remain only partly understood. Here, we explore cochlear morphology across 101 extant and fossil species of the mammalian grand order Euarchonta using micro-computed tomography, three-dimensional geometric morphometrics and phylogenetic comparative analyses. We find substantial shape variation across taxa, likely driven by an interplay between evolutionary history, morphological constraints and potentially functional demands, although these remain difficult to interpret. Evolutionary models suggest the rate of cochlear shape evolution was heterogeneous, with some lineages showing particularly high rates, likely linked with adaptive selection pressures (e.g. tarsiers, *Cercopithecus*). Ancestral state reconstructions reveal lemuriforms retain the ancestral strepsirrhine cochlear shape—conical with around 2½ turns—while lorisiforms exhibit a derived cylindrical cochlea with increased coiling. The highly coiled cochlea of tarsiers reflects cranial constraints and functional demands, particularly for high-frequency hearing. In anthropoids, platyrrhines nest within catarrhine variation. Among the latter, cercopithecins trend towards increased coiling, whereas colobines and hominoids retain less coiled shapes. Finally, while body size has little effect on cochlear shape, its interaction with cochlear length predicts the number of turns, supporting the theory that cochlear coiling enabled the development of a longer basilar membrane within a small petrous space.

## 1. Introduction

The study of biological shape dates back to early natural philosophy, from Aristotle’s writings to Leonardo da Vinci’s anatomical drawings. While these works already included precise measurements, mathematics was only integrated into morphological studies in the nineteenth century with the advent of morphometrics, formalized by biologists like D’Arcy Thompson [1]. These methods were further refined over time, allowing biological shapes to be studied in an evolutionary context. Three-dimensional (3D) shape analyses have further enhanced our understanding of complex biological structures, enabling studies of variation in function, phylogeny and overall bauplan. In this regard, the cochlea—the hearing organ of therians, characterized by at least one complete turn and the loss of the lagena macula [2]—is among the most distinctive mammalian structures.

The functional significance of the cochlea’s spiralling form has long intrigued biologists. Therians have longer basilar membranes (BMs)—housed within the spiral bony cochlear canal—than any other vertebrate clade, enabling them to hear a broader frequency bandwidth [3]. While the auditory benefits of BM elongation are relatively well understood, the role of cochlear coiling—and overall shape—remains debated. The prevailing view suggests coiling enabled the longer BM to develop while avoiding extensive changes to petrosal bone morphology and ensuring efficient innervation to receptor cells [2,4,5]. However, alternative explanations have been proposed, and an increased number of turns has been linked to both enhanced low-frequency perception [6–8] and higher-frequency hearing limits [9–11]. These studies often used the number of turns and measures like length and angles to describe the cochlea’s coiled shape. Some linear dimensions, such as cochlear volume [12,13], BM length [8,14,15] and width [16,17], show significant correlations with auditory parameters. Yet, recent findings added a new layer of complexity, revealing that taxa with similar body sizes and BM lengths can have differently shaped cochleae, leading to variations in hearing abilities [8]. In fact, an increase in cochlea length can be attributed to an extended hearing range, increased space per octave (improving resolution) or to both [2,18]. Thus, interpretations based solely on cochlear length remain approximate, and differences in octave distribution must be considered when comparing taxa, although these cannot be estimated from the bony cochlea alone.

Due to its complex morphology, particularly its variation in the number of turns, quantifying cochlear shape through linear parameters and classic morphometrics methods has presented methodological challenges and limitations. In the case of geometric morphometric analyses, landmarks are often slid to minimize bending energy and better capture shape variation [19]. However, this approach is not ideal for cochlear morphology and can introduce artefacts, such as artificially creating non-existent ‘loops’ along the curve defined by cochlear landmarks [8]. Additionally, cochlear shape analyses are often affected by the ‘horseshoe effect’, a phenomenon observed in ordination techniques applied to serial data [20]. This effect distorts the distribution of specimens in two-dimensional shape space, producing an arch-like pattern, particularly when the number of turns varies greatly [21,22].

To explore the evolutionary origins of the extensive morphological diversity observed in the therian cochlea [3,8,10,15], here, we focus on the shape of the euarchontan cochlea (Scandentia, Dermoptera and Primates), with an emphasis on primates. This species-rich and morphologically diverse group spans a wide range of body sizes and has a well-documented evolutionary history (electronic supplementary material, figure S1). Furthermore, primates provide a crucial framework for understanding human cochlear evolution, offering insights into the adaptations, developmental patterns and biomechanical constraints that have shaped *Homo sapiens*. Additionally, the functional morphology of the primate ear has been extensively studied, providing a strong foundation for inferring auditory capabilities in fossil specimens [11,12,23,24]. The cochlea is especially valuable for such research, as it is housed within the dense petrous bone, making it more resistant to taphonomic damage and more frequently preserved in the fossil record than other skeletal structures [25], yet it has rarely been examined with 3D geometric morphometrics [21,26], and its integration with modern phylogenetic comparative methods remains uncommon [8,23].

Here, we introduce a completely new geometric morphometric protocol to address the challenges in cochlear shape analysis (i.e. loops and horseshoe effect). Furthermore, our study aims to (i) describe shape variation in the euarchontan cochlea, (ii) assess its relationship to morphological and functional characteristics, and (iii) evaluate the role of allometry in shaping this variation. Additionally, we (iv) examine both phylogenetic and non-phylogenetic patterns and (v) investigate the mode and tempo of cochlear evolution in Euarchonta.

We hypothesize that cochlear shape variation is influenced by phylogenetic relationships, with distinct morphotypes emerging across euarchontan lineages, and by allometry, with shape covarying with body size and coiling constrained by the interaction between BM length and body size. We further hypothesize that evolutionary dynamics differ among lineages, reflecting distinct selective pressures and morphological constraints.

From these hypotheses, we predict that (i) closely related species will exhibit more similar cochlear morphologies than distant ones, (ii) cochlear shape will correlate with body size in accordance with allometric scaling, (iii) the degree of coiling will covary with BM length relative to body size, and (iv) cochlear evolution will follow different models of trait evolution depending on the lineage, with varying rates of morphological change that reflect different evolutionary scenarios.

## 2. Methods

### 2.1. Sample

In this study, we analysed the cochleae of 101 species (electronic supplementary material, figures S1), including 92 crown primates, 3 plesiadapoids (stem primates), 2 dermopterans and 4 scandentians. Our primate sample comprises 69 haplorrhines and 23 strepsirrhines. Among haplorrhines, we included 3 extinct omomyiforms, 2 tarsiiforms, 44 catarrhines and 20 platyrrhines. The strepsirrhine sample includes 3 extinct adapiforms, 8 lorisiforms and 12 lemuriforms. A total of 16 extinct species were part of this study, including one stem haplorrhine, three platyrrhines, three lemuriforms, three omomyiforms, three adapiforms and three plesiadapiforms. Each species was represented by 1–10 skulls (*n* = 176, median: 1, mode: 1).

The skulls of all extant species and some extinct ones are housed in several institutions (see electronic supplementary material, table S1). However, most fossil samples were obtained from MorphoSource (www.morphosource.org). Skulls of extant species were scanned at the Max-Planck Institute for Evolutionary Anthropology (Leipzig, Germany) using a Bruker™ SkyScan 2211 X-ray Nanotomograph. Voxel dimensions of the resulting scans ranged between 8.4 and 27 µm, while those for externally scanned fossil specimens ranged between 22 and 125 µm. The scans were processed to produce 3D surface reconstructions using Avizo Fire 9™ (3D Visualization And Analysis Software | Thermo Fisher Scientific - UK) by manually adjusting thresholds to isolate the bone from surrounding air and sediment. The ‘Isosurface’ module was then used to compute the 3D surfaces, which were stored in ‘.stl’ format. Following a published protocol [27], non-relevant structures were removed using the ‘Surface Editor’ tool. When digital extraction of the ear was not possible, the cochlea was reconstructed through manual segmentation, performed slice by slice across the dataset. The right ear was analysed when available; otherwise, the left ear was mirrored.

Adult body mass data were obtained from the PanTHERIA database, which reports singular species-level values [28]. The phylogenetic tree for extant species, including divergence times, was obtained from TimeTree [29] (http://www.timetree.org/), with fossil taxa added manually as follows: Plesiadapiformes as stem primates [30], adapiforms as the sister group to extant strepsirrhines [31] and omomyiforms as haplorrhines and sister group to tarsiers and catarrhines—though they may form a paraphyletic group [32]. Among platyrrhines, *Dolichocebus gaimanensis* and *Homunculus patagonicus* were initially considered stem platyrrhines [33], though the former is now considered closely related to modern Cebinae [34]; *Tremacebus harringtoni* was placed as part of Aotinae [35]. The phylogenetic position of fossil lemurs follows specialized literature [36], while *Aegyptopithecus* is considered a stem catarrhine [31].

### 2.2. Geometric morphometric analyses

A single researcher (J.d.R.) digitized landmarks and semi-landmarks for all cochleae in Avizo following a previously established protocol [8], where three fixed landmarks and an arbitrary number of semi-landmarks approximate the BM’s position (electronic supplementary material, figure S2). The only modification was resampling of semi-landmarks to 67 instead of 127, using the ‘equidistantCurve’ function of the ‘Morpho’ package [37] (v2.12) in R 4.4.1 [38]. For species with multiple individuals, their shapes were first aligned via generalized Procrustes analysis. The mean shape per species was then calculated as the landmark-wise average of these aligned configurations, using the ‘mshape’ function of the ‘geomorph’ [39] R package.

Geometric morphometric analyses allow for the study of shape independent of size, position and orientation [19]. Most analyses were conducted using the ‘Morpho’ and ‘geomorph’ packages. However, we developed a novel method to slide semi-landmarks, which addresses challenges that arise when analysing species where cochlear turns vary greatly. A key issue is the formation of unnatural ‘loops’ in cochleae with fewer turns. These artefacts occur when sliding algorithms distribute an equal number of landmarks across homologous cochlear turns, despite differences in turn numbers. With the total number of landmarks fixed and the first, second and last constrained, variation in turn number can cause landmarks to form loops in simpler cochleae. To address this, we initially considered equidistantly sampled landmarks without sliding, which prevents loops but artificially increases shape variation due to unoptimized bending energy. Instead, we developed ‘necklace sliding’, where landmarks slide along the cochlear curve like beads on a string (electronic supplementary material, methods S1 and text S1).

To explore shape differences, we conducted a principal component analysis (PCA) on the covariance matrix of Procrustes shape coordinates, which reduces dimensionality by identifying principal components that explain the most shape variation. As noted before, applying ordination techniques, such as PCA, to serial data can produce an artefact known as the ‘horseshoe effect’. In addition to the ‘necklace sliding’, as well as to complement our assessment of cochlear shape variation, we use adaptations of conventional PCA (phylogenetically aligned component analysis (PACA) and phylogenetic PCA (Phy-PCA); see below).

### 2.3. Cochlear dimensions

Landmark coordinates were also used to compute the length and number of turns of the cochlea in R. To calculate the length, we summed the distances between consecutive landmarks, starting from the round window and ending at the cochlear apex. For the number of turns, we developed an R script that allows very precise calculation (electronic supplementary material, methods S2 and text S2).

## 3. Phylogenetic comparative analyses

### 3.1. Phylogenetic signal

Closely related species exhibit greater similarity due to shared evolutionary history [40]. Therefore, these phylogenetically structured data violate the assumption of statistical independence among observations. To address this, we employed phylogenetically comparative methods.

Phylogenetic signal assesses how shared ancestry accounts for variation among related species. We used Blomberg’s K [40] to evaluate phylogenetic signal in the first three principal components of cochlear shape and its multivariate counterpart, Kmult [41], for the Procrustes coordinates. K or Kmult values near 0 indicate little to no phylogenetic signal; values around 1 suggest variation matches Brownian motion expectations, and greater than 1 imply greater similarity than predicted by Brownian motion. K and Kmult were estimated in R with the ‘phylosig’ and ‘physignal’ functions from ‘phytools’ [42] (v2.3-0) and ‘geomorph’ packages.

For the shape analyses, two adaptations of conventional PCA were used, PACA and Phy-PCA, which incorporate phylogenetic information in distinct ways. In PACA [43], the first components reflect the axes in shape space that best align with phylogenetic signal. In Phy-PCA [43,44], the first components are designed to minimize the influence of phylogenetic signal. These methods provide complementary perspectives for interpreting morphological variation in cochlear shape, highlighting the effects of shared ancestry and functional adaptations. Both were performed in R with the ‘gm.prcomp’ function of the ‘geomorph’ package

### 3.2. Allometric effect

To examine correlations between cochlear shape components and explanatory variables (e.g. body mass, centroid size, cochlea length and number of turns), we used phylogenetic generalized least squares (PGLS) [45] regression models in R, using the ‘pgls’ function from ‘caper’ package [46] (v1.0.3). This incorporates phylogenetic information into the regression error term, assuming Brownian motion trait evolution. Maximum likelihood Pagel’s lambda was used to transform tree branch lengths, accounting for phylogenetic signal variation.

We also performed regressions between Procrustes coordinates and explanatory variables, by fitting PGLS models to each principal component of shape, calculating maximum likelihood Pagel’s lambda for each model and computing a weighted average lambda using the percentage of shape explained by each principal component. This average lambda was used to inform the ‘procD.pgls’ function (geomorph package) to modify the tree branch lengths to reflect phylogenetic signal.

To examine whether the number of cochlear turns depends on the available space in the petrous bone for a given cochlear length, we performed a PGLS regression, using body mass (as a proxy for petrous bone size) and cochlear length as interacting variables. This allows us to assess not only the main effects of each variable but also whether the effect cochlea length has on the number of turns depends on body mass.

To account for the increased risk of false positives due to multiple comparisons, we applied *p*-value adjustments using the false discovery rate method. This method controls the proportion of false discoveries, ensuring that our significant results are more likely to reflect true effects rather than random chance. This was performed with the ‘p.adjust’ function of the ‘stats’ package in R.

### 3.3. Evolutionary models and ancestral state reconstructions

To investigate the mode and tempo of cochlear shape evolution in primates and predict ancestral cochlear morphology, we began by fitting several evolutionary models to the first three principal components derived from a standard PCA of cochlear shape, as well as to the number of cochlear turns. Tested models included Brownian motion, early burst (EB), Ornstein–Uhlenbeck and three different branch-length transformations: Pagel’s λ, Pagel’s κ and Pagel’s δ. We compared the Akaike information criterion (AIC) for each model to identify the best fit for each principal component and the number of turns.

To account for potential shifts in the rates of cochlear shape evolution, we first modified the phylogeny by adjusting the original branch lengths according to the best-fit model previously obtained for each of the first three principal components and the number of turns. We then applied a BM model to these modified phylogenies, allowing for up to 10 evolutionary rate shifts across the entire tree, in either individual branches or clades. The best models were selected by comparing their AIC scores, and branch lengths were further adjusted to incorporate any rate shifts detected.

The final branch lengths, accounting for these evolutionary models and rate shifts, were used to reconstruct ancestral states for the first three principal components and the number of cochlear turns. Evolutionary models were fitted using the ‘motmot’ package [47] (v2.1.3) in R, while ancestral state reconstructions were performed with the ‘phytools’ package. Rate shifts were identified using a modified version of the ‘summary.traitMedusa’ function from ‘motmot’, as the original did not correctly attribute shifts to the appropriate branches or clades (see electronic supplementary material, methods S3).

## 4. Results

### 4.1. Principal component analysis of cochlear shape

**Figure 1.**
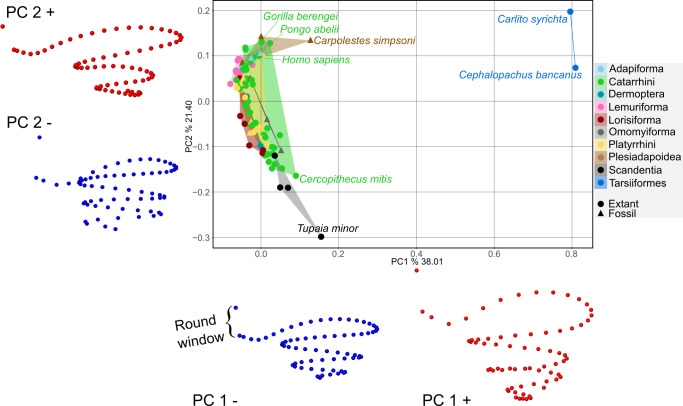
PCA of Euarchontan cochlear shape. The first two principal components (PCs) and associated shape changes towards negative (blue) and positive (red) PC scores.

The first three principal components together explain over 70% of cochlear shape variation (table 1, figure 1 and electronic supplementary material, figures S3 and S4). Tarsiers stand out for their highly coiled but conical cochleae, while scandentians and plesiadapoids represent opposite morphological extremes of cylindrical and highly coiled cochleae versus conical with fewer turns, respectively. Within anthropoids, catarrhines are more variable than platyrrhines, ranging from the conical cochleae of hominoids to the cylindrical forms of cercopithecins, with colobines in between. Strepsirrhine groups separate distinctly, with lemuriforms displaying consistently conical cochleae and lorisiforms more cylindrical morphologies with tighter coiling.

**Table 1.** Principal component (PC) axes of cochlear shape. Each axis is summarized by the main morphological changes associated with positive and negative scores, and by the taxa showing characteristic distributions along those axes. Together, the first three PCs explain 71.4% of total shape variation.

PC (% variance)	positive scores	negative scores	remarkable taxa distributions
PC1 (38.0%)	large round window; sharply curved first turn; loose coiling; high number of turns	small round window; softly curved first turn; tight coiling; low number of turns	highest scores: tarsiers followed by scandentians and *Carpolestes simpsoni* slight differences among other taxa along the lower scores: catarrhines: hominoids and colobines show negative scores; cercopithecines both negative and positive strepsirrhines: consistently negative scores, with the exception of Galagidae
PC2 (21.4%)	conical cochlea with fewer turns; round window at approximately 45° to modiolus	cylindrical cochlea with more turns; round window parallel to modiolus	highest scores: tarsiers and plesiadapoids lowest scores: scandentians catarrhines: hominoids show high scores; cercopithecins concentrate on low scores but are highly diverse; colobines plot intermediate strepsirrhines: lemuriforms show exclusively high scores, while lorisiforms are more diverse but tend to lower scores
PC3 (12.0%)	wide first turn oriented perpendicular to modiolus; tightly stacked turns	narrow first turn oblique to modiolus; loosely stacked turns	anthropoids show high scores lemuriforms show variable scores despite consistently high PC2 adapiforms plot consistently around zero scandentians, dermopterans, lorisiforms, plesiadapoids and omomyiforms show low scores

### 4.2. Main factors affecting cochlear shape

#### 4.2.1. Phylogenetic signal

Kmult’s estimated value was 0.41 (*p* = 0.001), indicating significant but moderate phylogenetic signal for the overall shape. For individual components, PC1 showed *K* = 1.21 (*p* = 0.001), suggesting closely related species resemble each other more than expected under Brownian motion. PC2 and PC3 had lower but significant signals (*K* = 0.40 and 0.78, *p* = 0.001 for both). The high *K* value for PC1 is likely driven by the separation of the two tarsiers from the rest of the taxa. In fact, by excluding tarsiers, Kmult decreased to 0.32 (*p* = 0.001) and the estimated *K* values for PC1, PC2 and PC3 were 0.35, 0.78 and 0.47 (*p* = 0.001 for all), respectively, meaning that species differed more in shape aspects than expected under stochastic evolution.

Along the first component of the phylogenetically aligned principal components (92% of the phylogenetic signal), negative values correspond to fewer turns and a round window running parallel to the first turn, while positive values indicate more turns and a round window perpendicular to the plane of the first turn (electronic supplementary material, figure S5). High PC2 scores indicate a conical modiolus, while low scores indicate a tubular one. Tarsiers and *Carpolestes simpsoni* show similarly high scores despite having the most and least turns, respectively. This might indicate the extinct plesiadapid displays a particularly elongated modiolus for their number of turns. The moderate phylogenetic signal suggests limited phylogenetic influence on cochlear morphology, and the species’ relative positions in the PACA partially resemble those of the conventional PCA. Some sister taxa remain unseparated (e.g. haplorrhines and strepsirrhines, platyrrhines and catarrhines), while others are distinguished (e.g. anthropoids and tarsiiformes, lemuriforms and lorisiforms). This indicates that while cochlear shape shows homoplasy and synplesiomorphy, it can distinguish between sister clades at certain taxonomic levels.

#### 4.2.2. Non-phylogenetic variation

**Figure 2.**
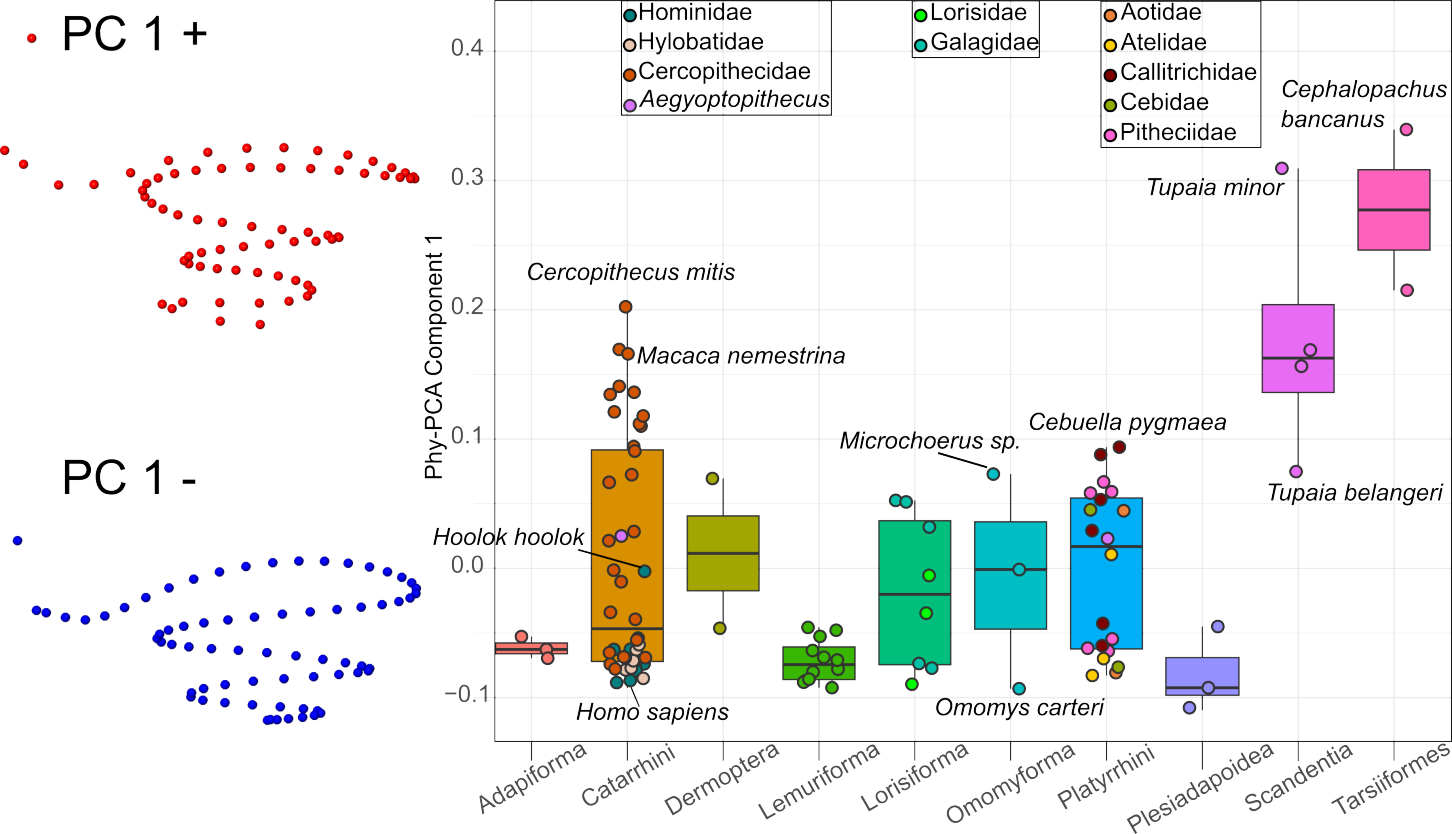
Boxplot of the first phylogenetic principal component. The first component is shown with associated shape changes towards negative (blue) and positive (red) scores.

The Phy-PCA in figure 2 offers a different perspective. Focusing on the first component—independent of phylogenetic signal—allows for the identification of non-phylogenetic factors, such as ecological variables or morphological constraints, driving morphological variation. However, the moderate phylogenetic signal in our shape variables means that this analysis does not diverge substantially from conventional PCA results.

The first component of the Phy-PCA accounts for 23% of shape variance independent of phylogeny. Negative values indicate fewer turns, a round window more parallel to the first turn that runs at an oblique angle to the modiolus and a narrow last turn. Positive values correspond to more turns, a round window oriented perpendicularly to the first turn, which runs perpendicular to the modiolus, and a wider last turn. Tarsiers and certain scandentians, platyrrhines and catarrhines diverge towards positive scores, indicating morphological variation linked to non-phylogenetic constraints. However, not all catarrhines show this divergence: only cercopithecins exhibit higher scores, while colobines and hominoids remain predominantly negative. No clear family distribution patterns are evident in platyrrhines, suggesting that non-phylogenetic factors significantly shape the cochlea in this group.

#### 4.2.3. Allometry

The influence of allometry on cochlear shape was tested using PGLS regressions between shape and size-related variables (electronic supplementary material, table S2). Multivariate regressions of Procrustes coordinates against centroid size and body mass showed weak but significant correlations (*R*² = 0.0412, *p* = 0.036; *R*² = 0.0343, *p* = 0.036). However, no individual principal components significantly correlated with size variables, confirming a minimal allometric effect on the main cochlear shape variation.

#### 4.2.4. Cochlea length and number of turns

Multivariate regressions between Procrustes coordinates and both cochlea length and number of turns yielded significant results, indicating an association between cochlear shape, length and coiling. However, the correlations were relatively weak (electronic supplementary material, table S3; *R*² = 0.0601, *p* = 0.006 for cochlea length; *R*² = 0.1035, *p* = 0.004 for number of turns).

Among shape principal components, only PC2 showed a strong correlation with both cochlear length and number of turns, particularly the latter (*R*² = 0.07, *p* = 0.010; *R*² = 0.412, *p* < 0.001). Only the PC1 correlation with the number of turns was significant, but weak (*R*² = 0.076, *p* = 0.008).

In the scatterplot for PC2 and number of turns, most data points follow the PGLS regression line (electronic supplementary material, figure S6), except for tarsiers, which are clear outliers (note that removing them increases the PGLS *R*² to 0.55 (*p* < 0.001)). Despite their high number of turns, tarsiers’ PC2 scores resemble those of *C. simpsoni*, the species with the fewest turns. Shape projections (figure 1) suggest that fewer turns correlate with a conical cochlea, while more turns correspond to a tubular one. However, tarsiers uniquely exhibit a conical cochlea, unlike any other species with high turns. For PC1 and the number of turns (electronic supplementary material, figure S6), removing tarsiers renders the regression non-significant (*R*² = 0.043, *p* = 0.23). Scandentians, which have the second highest number of turns after tarsiers, share high PC1 scores with *C. simpsoni*, which has the least coiled cochlea. This suggests PC1 may not be linked to the number of turns but instead reflects shared features like a sharper first-turn angle.

#### 4.2.5. Body mass–cochlea length interaction and number of turns

**Figure 3.**
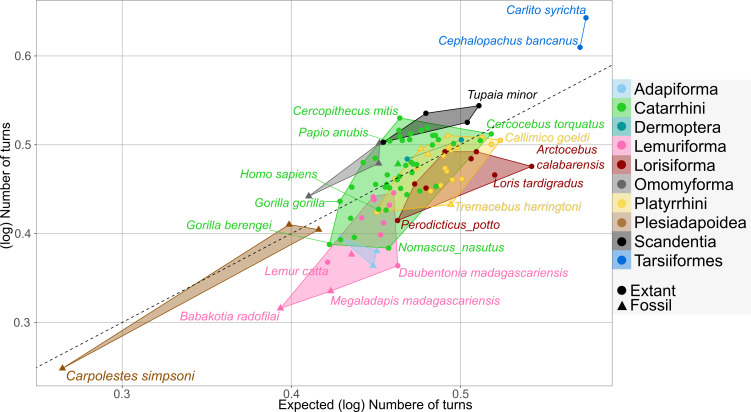
Scatterplot of the expected number of turns—predicted from the interaction between body mass and cochlea length—and the observed number of turns. A regression line with a slope of 1 and an intercept at the origin is provided as visual assistance.

The interaction between body mass and cochlea length shows a strong correlation with the number of turns (*R*² = 0.6, *p* < 0.001, *λ* = 0.816). Figure 3 illustrates actual versus fitted number of turns values. While most taxa align with expectations, tarsiers, scandentians, cercopithecids and omomyiforms exhibit more turns than predicted, though the last remain closer to expected values. In contrast, all strepsirrhines, adapiforms, most hominoids and platyrrhines show less coiling than expected. Plesiadapiformes, adapiformes and dermopterans fall within expected ranges.

#### 4.2.6. Models of trait evolution and ancestral state reconstructions

**Figure 4.**
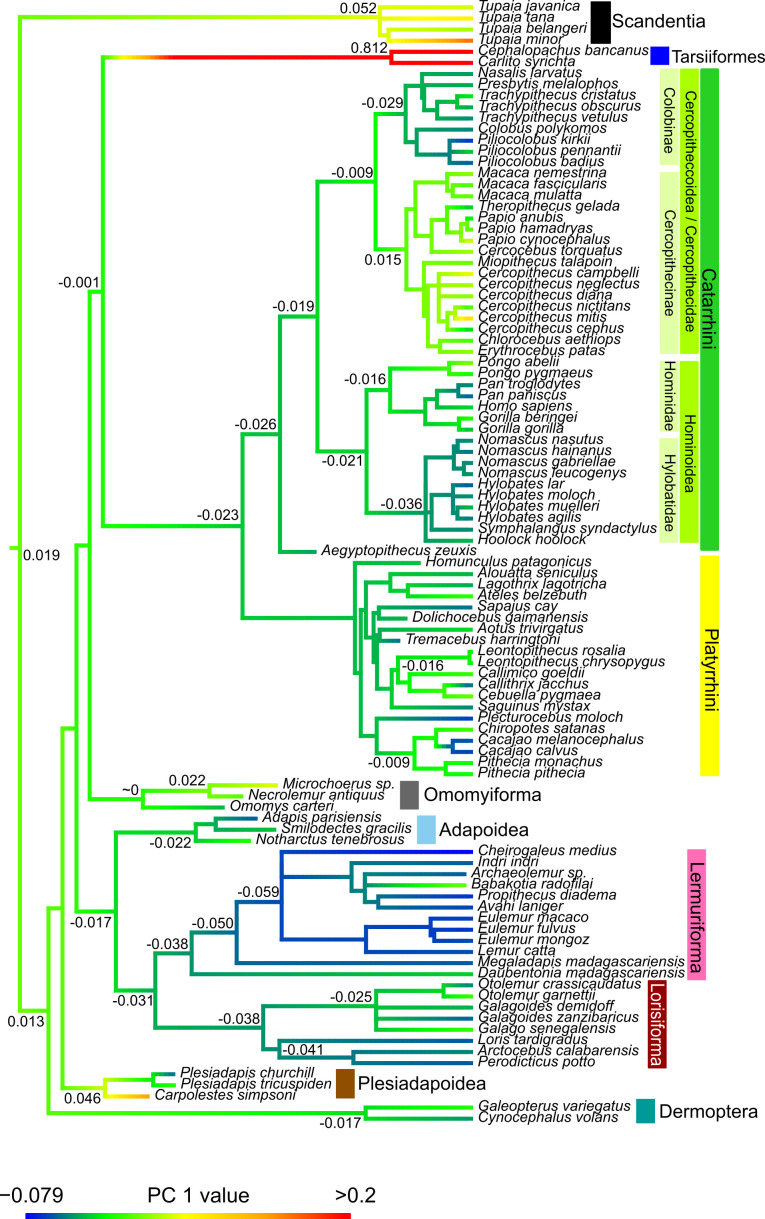
Ancestral state reconstruction of PC1 values. Exact values at selected nodes are provided.

For PC1, an EB model with five rate shifts provided the best fit. The initial rate of evolution ($\sigma_{0}^{2}$) was estimated at 1.24 × 10^−3^, with a rate change parameter (*r*) of −2.79 × 10^−2^ (95% confidence interval (CI): −4.17 × 10^−2^ to −1.46 × 10^−2^). This model had a corrected AIC (AICc) of −306.2 and a maximum likelihood value of 156.2. Relative rate increases occurred in the branch leading to modern tarsiers, the branch leading to the *Cacajao* clade and in the *Cercopithecus* and *Papio* clades. In contrast, there was a single rate decrease in the clade formed by *Eulemur* and *Lemur*. The ancestral state reconstructions for PC1 (figure 4) reveal the following. Extant tarsiers’ cochlea morphology—characterized by high PC1 scores, larger round window, angular first turn and higher turn number—likely developed in their common ancestor, though precise timing remains uncertain without fossil data. The estimated anthropoid ancestor had low PC1 scores (small round window, gently angled first turn, few turns), features mostly retained in modern species except some platyrrhines and cercopithecids. Consequently, cercopithecine and select platyrrhine ancestors (Pithecidae) developed fewer turns and softly angled first turns. Most hominids retain the ancestral anthropoid condition, with *Pan* as an exception. Gibbon ancestors developed lower scores, consistently preserved in extant taxa. The ancestral colobine independently regressed to lower scores, a feature preserved in extant species. Among omomyiforms, *Microchoerus* sp. and *Necrolemur antiquus* exhibit relatively high scores compared to *Omomys*, diverging from the ancestral group condition. Most strepsirrhines, including extinct Adapoidea, display low PC1 scores, supporting a low-score ancestral cochlea. Within strepsirrhines, lemuriforms and lorisiforms share similar ancestral scores, with a notable variation at the split between *Daubentonia*, which retains higher scores, and the ancestral lemuriform, which developed lower scores. Within lorisiforms, Galagoidea ancestors evolved higher scores, while Lorisoidea retained the plesiomorphic strepsirrhine shape. Scandentians have higher scores than most primates, potentially diverging from the euarchontan morphology towards more positive values, though fossil evidence is absent. The ancestral Plesiadapoidea had higher scores, but while *Carpolestes* developed even higher values, the ancestral *Plesiadapis* regressed to lower ones. Finally, extant dermopterans retain scores rather similar to those of the ancestor of colugos.

For PC2, Pagel’s *λ* model with three rate decreases in *Hylobates*, Lemuriforma and the *Chlorocebus-Erythrocebus* clade provided the best fit. The maximum likelihood *λ* estimate was 0.90 (95% CI: 0.76–0.97), with *σ*² = 1.62 × 10⁻⁴, log-likelihood of 131.95 and AICc of −257.7. The ancestral state reconstruction (electronic supplementary material, figure S7) shows that low PC2 scores, associated with tower-shaped cochleae, developed primarily in scandentians and likely their common ancestor. The euarchontan ancestor had a slight inclination towards a conical shape, similar to omomyiforms and most extant primates, except the ancestral lorisiform and ancestral anthropoid that have apparently converged with scores close to 0. Among catarrhines, colobines resemble the ancestral group condition, while cercopithecines show lower PC2 scores, indicating a derived tower-like cochlea. Hominoids regressed to higher scores, suggesting a more conical morphology, particularly pronounced in hominids contrasting with hylobatids. Platyrrhini diverged slightly from the ancestral anthropoid condition, showing great diversity at the tree tips towards lower values, although some Atelidae exhibit slightly higher values. Lemuriforms retain the ancestral strepsirrhine state with high scores, while lorisiforms developed lower scores. Within lorisiforms, the ancestral galagoid developed even lower scores, in contrast to the ancestor of lorisoids that remained similar to the group’s plesiomorphic state. Dermopterans diverged towards lower scores, though fossil evidence is lacking.

For PC3 ancestral reconstruction results, refer to electronic supplementary material, figure S8 and results 1.

#### 4.2.7. Ancestral state reconstruction of the number of turns

Ancestral state reconstruction of cochlear turn number (electronic supplementary material, figure S9) identified Pagel’s *λ* as the best-fitting model, with three rate increases occurring in *C. simpsoni*, *Nomascus* and the branch leading to tarsiers. The maximum likelihood estimate of *λ* was 0.97 (95% CI: 0.92–1.00), indicating a phylogenetic signal generally consistent with Brownian motion. The evolutionary rate (*σ*²) was estimated at 3.02 × 10⁻³, with a log-likelihood of 1.17 and AICc of 3.90. The unique morphology of *C. simpsoni* (1.75 turns) represents a derived trait, contrasting with the ancestral plesiadapiform condition (approx. 2.5 turns). Most other clades exhibit minimal divergence from ancestral states, though some exceptions exist. The lorisiform ancestor evolved slightly more turns than lemuriforms, which retain the ancestral strepsirrhine state. Similarly, hominoid ancestors underwent a slight reduction in the number of turns. Within Haplorrhini, tarsiers developed highly coiled cochleae, while most catarrhines and platyrrhines retain the group’s ancestral conditions. The ancestral cercopithecine diverged by developing more turns relative to colobines. Dermopterans show a slight divergence from the ancestral euarchontan state towards more turns, contrasting with scandentians’ derived, highly coiled cochleae. However, the absence of fossil dermopterans and scandentians in the dataset complicates ancestral state estimation.

## 5. Discussion

Our analyses reveal that in euarchontans, cochlear shape likely reflects a complex interplay between anatomical constraints, shared ancestry and potentially function, which is difficult to unravel. Although our analyses did not include audiometric data, previously published audiograms provide a valuable comparative framework. By contrasting our morphological results in the light of previous auditory research, we can cautiously interpret certain cochlear features in terms of their potential auditory function. To achieve this, we thoroughly described the diversity of the euarchontan cochlea, which ranged from only 1.7 turns in the extinct plesiadapid *C. simpsoni* to the highly derived cochleae of modern tarsiers with over four turns. While several higher-order taxa show substantial shape overlap, great diversity in shape was also observed within these groups.

### 5.1. Cochlear shape variation and function

Tarsiers stand out in our analyses, exhibiting a combination of a large round window, a sharp angle in the initial turn and loose coiling with more than four widely spaced turns. This unique configuration may result from a combination of adaptive pressures: tarsiers have exceptional auditory capabilities, particularly in producing vocalizations up to 70 kHz and detecting high-frequency sounds up to 90 kHz [48], some of which are produced by their prey [49]. However, spatial constraints on the cranial base cannot be ruled out (see below).

The extinct omomyiforms exhibit cochlear shapes similar to extant lorisiforms—with somewhat cylindrical cochleae, small round windows, a softly curved and narrow first turn, a loose stacking and a high number of turns—pointing towards similar auditory functions potentially linked to their hypothesized nocturnal behaviour [50]. A high number of turns and loose coiling are generally associated with a broader hearing range [6,7,14,51]; the extension of the hearing range towards the higher frequencies would have been advantageous at night by improving sound localization [52] and detection of prey or predator-related sounds in low-light conditions [48,49]. Adapoids, conversely, show cochlear structures that cluster more closely with those of lemuriforms. However, differences in hearing between lemuriforms and lorisiforms are reportedly small, and lemurs might only have marginally better high-frequency hearing, which would be advantageous for their more complex social habits, as reported by previous research [53–55]. For example, high-frequency communication bands offer ‘private’ channels less masked by low-frequency environmental noise, enabling species to develop a wider array of calls for alarm, mating and group coordination without interference [54,56]. Nonetheless, differences in cranial integration may also provide an explanation, as lorisiforms have larger orbits than lemuriforms due to their nocturnal habits [31], which would also explain the similarity between the former and omomyiforms (see below). However, available audiogram data for these groups are very limited, and a definitive explanation for these morphological differences cannot be provided for now. There are also no available audiogram data for the two dermopteran species, but they display a cochlear morphology that is similar to most primate groups, particularly to lorises. This suggests some overlap in auditory function, possibly due to shared arboreal and nocturnal behaviours. A study on the morphology of their middle ear, however, showed that colugos exhibit a primitive eutherian auditory region, which is associated with enhanced perception of low- to mid-frequency sounds [57].

Among anthropoids (platyrrhines and catarrhines), cochlear morphology shows broad similarity, with considerable overlap in overall cochlear shape. Catarrhines, however, display greater variation, which may reflect their broader range of body masses [58], geographic distribution and habitats [31]: in closed environments like rainforests, reverberation and vegetation attenuate high frequencies quickly, but these remain useful for short-range communication, while low-frequency sounds travel farther yet may be masked by background biotic noise. In open environments like savannas, wind and atmospheric turbulence disrupt high frequencies, favouring low-frequency signals and perception [55,59]. Within catarrhines, hominoids trend towards a more conical cochlea with around 2.7 turns, while cercopithecines exhibit mostly cylindrical shapes—particularly colobines—with around three turns. Notably, despite the human unique capacity for spoken language, our bony cochlea falls well within hominid variation in the shape space produced by our analysis and shows no signal of any derived feature pointing to any form of hearing specialization, mirroring the broadly similar hearing capacities when compared to chimpanzees [60]. Again, the functional implications of cochlear shape remain challenging to assess due to the lack of hearing data for the majority of species [61]. However, based on morphological traits and the available audiograms of several cercopithecins, humans and chimpanzees, it is generally assumed that hominids have better low-frequency hearing than cercopithecoids [62], and by extension hylobatids—which cluster morphologically with monkeys rather than with great apes [63]—cercopithecins have a better high-frequency sensitivity. Future research is needed to confirm this assessment.

Despite the gap in available audiometric data, the results of our Phy-PCA (figure 2) reinforce the idea that variation in cochlear shape could be related to function; the allometric signal in our sample was weak, and thus the species distribution in the Phy-PCA likely reflects either functional cochlear features or spatial constraints. Tarsiers, scandentians and, in particular, some groups of platyrrhines and cercopithecids exhibit a higher number of turns, a round window oriented perpendicularly to the first turn with the first turn being oblique to the modiolus, and a wider last turn. Since Phy-PCA removes the influence of phylogeny from the analysis, taxa whose morphology is most deviating could harbour features associated with adaptive responses to either ecological (e.g. forest versus open habitat) or sensory demands, or to morphological constraints. In catarrhines, for example, this divergence is primarily observed in cercopithecins rather than colobines or hominoids. Although this study focuses exclusively on cochlea morphology, this finding indicates that colobines and great apes could share similar audiometric features.

The observation that Old World monkeys have more cochlear turns than great apes would challenge earlier findings that a higher number of turns was associated with better low-frequency hearing, particularly in euarchontoglires [8]. Tarsiers add to this complexity by having both the highest number of turns and the best high-frequency hearing among primates [48]. Since some studies associate more coils with improved low-frequency hearing [6–8,64] and others with improved high-frequency hearing [2,4], the functional significance of cochlear coiling remains unclear. One explanation may lie in the relationship between the number of turns and the length of the cochlea, but support for such a relationship is not very strong in the literature so far [7,65] (but see below). Evidence for a relationship between cochlear length and hearing is also limited, but it is generally accepted that a longer cochlea correlates with both better low-frequency hearing [8,14] and worse high-frequency hearing [15]. Furthermore, the width of the BM—particularly at the apex—strongly influences hearing [16], and wider apical membranes are associated with improved low-frequency sensitivity, as documented in large mammals such as elephants and mysticetes [3,17]. Although BM width can often be approximated from the gap between both cochlear bony laminae [16]—though with some uncertainty—the secondary bony lamina is greatly reduced or entirely absent in most anthropoids [66]. Thus, our functional interpretations rely primarily on cochlear length and shape and should be considered with this limitation in mind. In this sense, studies have shown that cochlear shape can indeed influence auditory function, and similar results were found for middle ear structures [6,67]. Based on our findings, it is likely that cochlear coiling may have multiple functional roles depending on the species, but more research needs to be conducted in order to fully understand its implications.

### 5.2. Morpho-spatial constraints on cochlear shape

Although it is tempting to assign a functional significance to the observed variation, part of it may also reflect spatial constraints imposed by other cranial structures, complicating direct comparisons of shape and function across samples with a great body size range like ours (but see [8]). In this sense, the cochlea of tarsiers may reflect functional demands, though their enlarged orbits might also influence skull organization, including cochlear orientation [68]. This could explain the unique angle between the round window and the bony cochlea’s first turn. Furthermore, tarsiers exhibit a conical cochlea with radii strongly decreasing towards the apex—a feature otherwise restricted to euarchontans with fewer turns. This morphology could reflect space-saving needs achieved by reduced turn radii, tied to their distinct cranial organization. In contrast, taxa like scandentians and some cercopithecids with a high number of turns display cylindrical cochleae where radii barely decrease, which could indicate fewer spatial constraints. At the opposite extreme, *C. simpsoni* (a Palaeocene plesiadapoid) shows an exceptionally low cochlear turn count (1.7), particularly given its cochlear length. This morphology parallels that of some similarly sized marsupials [8] and may imply a reduced hearing range through low-frequency octave loss, as seen in such groups [6,7,14]. However, *Carpolestes*’ short cochlea relative to body size—contrasting with tarsiers—directly explains its low turn count.

Furthermore, our results show only a weak allometric signal, suggesting body size alone does not strongly constrain cochlear shape evolution. This aligns with studies in platyrrhines [23], catarrhines [62] and euarchontoglires [8]. The weak cochlear shape–body size relationship in primates may allow greater plasticity in adapting auditory structures to ecological niches, communication needs, or cranial base organization. However, our results also show that body size and cochlear length interaction strongly determines the number of turns. Therefore, we provide what is, to our knowledge, the first empirical support for the theory that inner ear coiling enabled the development of longer BMs without major petrosal morphology changes [4,14]. These findings suggest that in smaller euarchontans—and potentially in small-bodied mammals in general—increased cochlear coiling is necessary to accommodate longer BMs within the spatial constraints of the petrosal. In contrast, this constraint appears to be alleviated in larger species, allowing for longer BMs without the need to increase cochlear coiling. Additionally, several euarchontan taxa exhibit exceptionally coiled or uncoiled cochleae relative to the predicted values obtained from the interaction between body mass and cochlear length: tarsiers, scandentians, cercopithecids and omomyiforms display more turns than the interaction predicted, possibly reflecting functional adaptations. Conversely, strepsirrhines and most hominoids show reduced coiling, which some studies link to improved high-frequency hearing [8,51,64], although it could also imply a reduced hearing range [6,7,14]. The low cochlear turn count of *C. simpson* (1.7) closely matches predictions for its body mass and cochlear length. This suggests *Carpolestes*’ uniqueness lies in its short cochlear canal, resulting in its reduced number of turns. This contrasts with tarsiers, which exhibit both longer cochleae and more turns than predicted for their body mass (electronic supplementary material, figure S10). This raises the question of whether *Carpolestes*’ morphology represents a derived specialization or retention of the ancestral therian condition. Ancestral state reconstruction (discussed further below) supports a derived reduction, reverting to a cochlear shape similar to the ancestral therian state of just under two turns [65].

### 5.3. Cochlear shape evolution in the light of phylogenetic constraints

Apart from functional and spatial constraints, evolutionary relationships have also influenced euarchontan cochlear shape. In other mammalian clades, such as echolocating bats [51] and infrasound-specialist cetaceans [64], cochlear coiling has been shown to reflect both phylogenetic relationships and functional adaptations related to their specialized auditory capabilities. Our analyses reveal that in euarchontans, considered hearing generalists, phylogeny plays a significant role in shaping cochlear morphology, as indicated by a significant but moderate phylogenetic signal.

Our results indicate that cochlear shape evolution is more complex than a Brownian motion model. Different aspects of cochlear morphology face varying selective pressures depending on the taxonomic group. For example, characters associated with PC1—such as the first turn’s shape and the round window’s relative size—fit an EB model with five rate shifts. This indicates that certain cochlear shape aspects underwent rapid morphological change, followed by a slowdown in some lineages. The observed rate shifts in *Eulemur/Lemur*, Tarsiiformes, *Cercopithecus*, *Papio* and *Cacajao* suggest potential selective pressures or evolutionary events influencing cochlear shape in these groups. Further research is needed to determine whether these shifts relate to ecological adaptations, changes in communication strategies, or other lineage-specific factors.

Among strepsirrhines, lemuriforms appear to retain the plesiomorphic cochlea state of strepsirrhines [69],—conical shape—as supported by the inclusion of fossil adapiforms. In contrast, the cylindrical cochlea of lorisiforms represents the derived condition, possibly reflecting adaptations related to their nocturnal and/or solitary lifestyle. As discussed above, differences in hearing between lemuriforms and lorisiforms are small; however, this could relate to differences in petrous and cranial integration. Similar patterns where lemuriforms retain more plesiomorphic traits have been observed in other aspects of strepsirrhine biology, such as birthing twins [70] or having a smoother cerebrum with fewer superficial folds [71].

The evolutionary reconstruction of cochlear shape within anthropoids reveals conserved and derived traits across lineages. The ancestral anthropoid condition, with a smaller round window, softly angled first turn and 2¾ turns in a shape between cylindrical and conical, is retained in many extant catarrhines, particularly colobines and hylobatids. Cercopithecins diverged towards the primitive primate shape, developing more turns, more angled first turns and cylindrical shapes early in their evolution. Hominids have slightly reduced turn numbers and developed a more conical cochlea, though diverging less from the ancestral condition than other anthropoid groups.

By comparing our results with those found for other anatomical structures, we can provide tentative explanations for the evolutionary patterns observed. For instance, in platyrrhines, aspects of brain shape better fit an EB model of trait evolution [72]. While our data support an EB model across euarchontans, no overall rate change is identified for all platyrrhines, only an increase in rate in the genus *Cacajao*. This suggests that the hearing organ may have undergone evolutionary changes independent from the brain, at least in New World monkeys.

Our findings that some cochlear shape characteristics underwent an EB in evolution provide a compelling basis for discussing evolutionary forces influencing inner ear morphology and euarchontan morphology more broadly. The EB model is rarely supported in morphological evolution and has been debated in evolutionary biology. While theory and fossils suggest EBs should be common, studies using only extant taxa often fail to support it [73]. Parts of our results, however, align with these predictions. For example, platyrrhine diversity may result from adaptive radiation, as suggested by several studies of morphological traits [23,74,75]. Additionally, the larynx, the primary sound-producing organ, was found to experience rapid early evolution in primates, especially compared to carnivores [76]. This evidence suggests both sound-producing and sound-perception organs of primates underwent significant early evolutionary changes.

An adaptive radiation may correlate with an EB model, but evidence suggests these concepts rarely align [73]. The contrast between our findings and those of other studies highlights primate evolutionary history’s complexity and underscores the need to study multiple biological traits to fully understand it. Future research could explore why some aspects of cochlear evolution differ from other traits of the euarchontan bauplan, particularly compared with other auditory structures like the middle ear and external ear. Comparative studies with other mammalian groups may also reveal whether this is specific to euarchontans or part of a broader pattern in mammalian auditory evolution.

## 6. Conclusion

Our study of cochlear morphology reveals a complex interplay between evolutionary history and other factors in the Euarchonta taxon. Notably, body size does not constrain cochlear shape evolution, allowing for greater adaptive flexibility in auditory structures. However, in contrast to shape, the number of cochlear turns is constrained by the interaction of body size and cochlea length. This is the first time empirical evidence has been provided to support the view that the cochlea developed as a space-saving feature. Furthermore, modelling cochlear shape evolution highlights the complexity of these processes, with different morphological traits—i.e. the first-turn angle or overall shape—fitting different evolutionary models. Nonetheless, we propose that among higher taxonomic levels—e.g. order or even family—the cochlea contains enough phylogenetic information to serve as a valuable tool for taxonomic classifications. Patterns in cochlear morphology—like the retention of ancestral traits in lemuriforms, the unique morphology of tarsiers, the derived features of the early stem primate *C. simpsoni* and the high evolutionary plasticity among anthropoids—offer insights into the processes shaping primate auditory systems. Future comparative studies could include more cochlear parameters that could not be included here—like BM width—and expand to other mammalian groups. More auditory data are also essential to further clarify the evolutionary forces driving auditory structure diversity.

## Data Availability

The ‘code&data_prims_OS.zip’ file contains the code and raw landmark data associated with the paper. The files are organized as follows: 1. Primates_cochlea_script.Rmd, an R markdown file containing the complete R code used for all statistical analyses and data processing presented in the paper and electronic supplementary material. 2. Necklace_sliding_function.RData + Supplementary_text_1.txt, an R workspace file that allows direct loading of the *necklace sliding* function into the R environment. The function is additionally provided in a text file. 3. Number_turns_function.RData + Supplementary_text_2.txt, an R workspace file that loads the cochlear turn calculation function directly into the R environment. The function is additionally provided in a text file. 4. Resample_primates.R, an R file containing the code used to resample the landmarks to 70. 5. Landmark_files, a folder containing 154 .tps files with the raw landmark coordinates from all extant species included in the study. Note that fossil specimens and any derivative data associated with them are not included. These data were obtained from MorphoSource and other institutional collections with restricted data-sharing policies. As such, we do not have permission to redistribute those files. The MorphoSource accessions used in this study include the following: https://n2t.net/ark:/87602/m4/M1949, https://doi.org/10.17602/M2/M35988, https://n2t.net/ark:/87602/m4/M33924, https://n2t.net/ark:/87602/m4/M34023, https://n2t.net/ark:/87602/m4/M34026, https://n2t.net/ark:/87602/m4/M36672, https://doi.org/10.17602/M2/M945, https://doi.org/10.17602/M2/M9341, https://doi.org/10.17602/M2/M9345, https://doi.org/10.17602/M2/M9347, https://n2t.net/ark:/87602/m4/M33831, https://n2t.net/ark:/87602/m4/M76092, https://doi.org/10.17602/M2/M110555, https://n2t.net/ark:/87602/m4/M77534, https://n2t.net/ark:/87602/m4/M64309, https://n2t.net/ark:/87602/m4/M64314, https://doi.org/10.17602/M2/M9339. Supplementary material is available online [77].
